# Enhancing Radiation Oncology Delineation Skills Through Simulation-Based Learning (SBL): A Five-Step Practical Approach Using the ADDIE Model

**DOI:** 10.1007/s13187-025-02637-8

**Published:** 2025-05-15

**Authors:** Hany S. Attallah, Rania Abdelghani, Omayma Hamed

**Affiliations:** 1 Radiation Oncology, AFCM, Cairo, Egypt; 2 Radiation Oncology, Maadi Military Hospital, Cairo, Egypt; 3 Dermatology, Armed Forces College of Medicine, Cairo, Egypt; 4 Faculty of Medicine for Girls, Al-Azhar University, Cairo, Egypt; 5 Medical Education, AFCM, Cairo, Egypt

**Keywords:** SBL, ADDIE model, Delineation skills, Radiation oncology

## Abstract

Simulation of radiotherapy treatment setup is an integral part of the daily radiation oncology practice. Hence, simulation-based learning (SBL) plays a critical role in radiation oncology resident training. Such practical training approach enhances the precision of tumor delineation and the organs at risk (OARs), thus, directly influencing treatment efficacy and patient safety. Our review explores the application of the ADDIE (analysis, design, development, implementation, and evaluation) model in developing and implementing an SBL program of delineation skills training for the radiation oncologists. In this review, we discussed the phases of the ADDIE model as follows: analysis to determine the skills gap, design to tailor the training program to incorporate the advanced technology methods, development targets creating practical case scenarios and assessment tools, implementation assures the real-time feedback and the model-based debriefing sessions, and finally evaluation phase that employs the Kirkpatrick model. The application of the ADDIE model in SBL emphasizes that the training aligns with the educational needs. This enhances the clinical performance, and consequently, improves patient outcomes in radiation oncology. Applying the ADDIE model in SBL achieves the educational needs in radiation oncology delineation skills training, which will lead to enhance the clinical performance.

## Introduction

Simulation-based learning (SBL) has become an integral component of advancing healthcare education, especially in disciplines like radiation oncology where precision is critical. The precise delineation of tumors and organs at risk (OAR) is paramount in radiation therapy. Because it has a direct impact on treatment efficacy and patient safety. Traditional training methods, such as didactic lectures with limited hands-on practice, often fall short in equipping trainees with the basic skills for precise tumor and OAR delineation [1, 2].

Research highlights a significant variability in delineation skills among trainees, which contributes to the potential treatment errors, e.g., under-dosing of the tumor or overdosing of the OARs. These facts call for the urgent need for enhanced training methods. SBL addresses this gap as it provides a controlled, risk-free environment where trainees can repeatedly practice and sharpen their skills. High fidelity and medium-fidelity simulations offer immersive experiences that bridge the gap between theoretical knowledge and practical application. Consequently, it will assure both the competence and confidence of trainees [3–5].

The ADDIE model (analysis, design, development, implementation, and evaluation) is a structured framework that is a widely used instruction, particularly in the development of educational programs that targets the learner needs. Our review synthesizes the application of the ADDIE model in radiation oncology SBL, drawing on a broad spectrum of literature to illustrate how this model enhances the training of delineation skills. By detailing the integration of ADDIE within SBL, this article aims to provide radiation oncologists and educators with a comprehensive understanding of effective educational strategies to foster technical proficiency and decision-making in high-stakes medical fields [6].

## Phase 1: Analysis

In this phase, we focus on revealing the performance gaps in delineation skills among radiation oncology trainees.

### Needs Assessment

The documented variability of delineation skills among trainees can lead to significant clinical risks, including tumor under-dosing or OAR overdosing. The traditional educational methods reported to fall short in addressing such complexities. SBL suggests a solution of this problem, by providing immersive and repetitive training environments that are enriched with feedback and debriefing. Such approach would significantly improve the skill acquisition and application [7, 8].

### Task Analysis

Trainees are required to perform the complex task of the precise delineation of tumors and OARs, using the advanced imaging technologies. This will need them to master the use of treatment planning software. Reflective practice is encouraged to integrate debriefing ad feedback [7, 8].

### Learner Analysis

During their initial training, the radiation oncologists had already acquired the basic theoretical knowledge. However, they lack the practical skills in delineating the tumor and the OAR. SBL program possess a solution to target the needs we reported through surveys and interviews [9]. Their needs included the inclusion of interactive learning and feedback-based instructional methods.

### Performance Analysis

Their initial OSPE assessment showed that the trainees often struggle with delineating the tumor and the OAR. That was most eminent in anatomically complex regions, e.g., head and neck region. The training aims to cater their proficiency in delineation, while developing critical analytical skills [10].

### Learning Objectives

Consequently, we decided the intended learning objectives (ILOs) of the program to be as follows: cognitive objectives: (1) to focus on empowering the learners with accurate identification of the anatomical landmarks on the imaging studies, e.g., CT and MRI, and (2) to understand the fundamentals of dose distribution, dose constrains, and the therapeutic ratio in radiation therapy, psychomotor objectives: (1) to demonstrate proficiency in using treatment-planning software, e.g., eclipse, and finally, behavioral objectives: (1) to adhere to safety protocols and contouring guidelines, in order to avoid neither the overdosing nor the under dosing [11].

## Phase 2: Design

In his phase, we focus on framing the SBL course so that it meets the identified needs and the decided ILOs (e.g., triangulation). Additionally, we assure that the used instructional materials support the delineation skill effectively.

### Learners

The attendees’ number is limited to a group of 10 junior radiation oncology residents, to allow personalized interaction with the facilitators. This group education setting enhances PAL, e.g., enables the trainees to benefit from diverse experiences shared among peers [12].

### Environment

The SBL training should be conducted in a radiation oncology department that is designated as a center of excellence. The equipment of the department should align with the currently used treatment planning software in the workplace of the candidates. Such setup would enhance the authenticity and applicability of the course [13].

### Methodology

Experts in SBL now acknowledge debriefing as a crucial process that connects theoretical learning with practical skill application. Research shows that the structured debriefing enhances clinical skills and, consequently, directly affects the patient care outcome. The SBL program’s methodology starts with a pre-simulation briefing that thoroughly prepares participants by explaining both the simulation objectives along with the functions of the software tools they will operate. The training briefing establishes clear trainee expectations and outlines clinical scenarios to ensure participants are thoroughly prepared [14]. The debriefing sessions employ the established frameworks, i.e., the GAS (gather, analyze, and summarize) model [15] and the PEARLS (promoting excellence and reflective learning in simulation) model [16].

### Resources Needs

The resources for applying an effective SBL would include (1) advanced delineation software like Eclipse or Pinnacle and (2) a robust infrastructure, i.e., internet access and smart screens. Human resources such as well-trained faculty are essential for delivering effective debriefing and feedback [8].

### Feedback

The use of feedback models, such as Pendleton’s model [17], would assure that constructive feedback is delivered with clarity. This aims to foster the supportive learning environment and encouraging continuous improvement in delineation skills.

## Phase 3: Development

This phase revolves around creating the tools of the course, i.e., case scenarios and task sheets. It also includes the development of assessment tools like OSPE rubrics that align with learning objectives.

### Pre-simulation Preparation

Essential simulation setup includes equipping workstations with advanced treatment planning software and updated imaging datasets. Preparation involves distributing detailed guides and protocols to ensure trainees understand their tasks and safety procedures before the simulation begins [17]. To guide the learners effectively, task sheets are prepared and distributed before the simulation begins, as shown in Table 1.

**Table 1:** Task sheet for delineation

Case no,	Case no. 1
Presenting complain	Change of voice over the last 3 months
Case description (e.g., patient history, medications, physical exam)	Mr. Ahmed M., a 55-year-old male, presents with a 3-month history of progressively worsening hoarseness, difficulty swallowing, and ear pain on the left side, accompanied by a recent 5-kg weight loss. A heavy smoker with a 20-pack-year history and moderate alcohol use, he has no significant family history of cancer. Physical examination reveals a palpable left cervical lymph node, and flexible laryngoscopy shows an irregular mass on the left vocal cord, extending to the anterior commissure. CT imaging confirms a 2.5-cm mass involving the left vocal cord with paraglottic space involvement and multiple left-sided cervical lymph nodes, the largest measuring 2 cm. He is diagnosed with Stage III laryngeal cancer (T3 N1 M0)
Task(s) for learner	Delineate the tumor Delineate the involved cervical LN Delineate the OAR: spinal cord, carotid arteries, parotids

### Hands-On Simulation Activity

Trainees engage in practical delineation tasks using patient datasets. Real-time feedback from faculty enhances learning, while PAL during collaborative contouring exercises fosters a supportive learning environment [12, 13, 18].

### Debriefing Session

During these sessions, facilitators guide trainees through a systematic evaluation of their actions, emphasizing the importance of critical thinking and self-assessment. For example, the PEARLS model integrates the targeted ILOs and conformational debriefing strategies to align with the complexity of the SBL and the reported learning needs of the trainees. Such approach is crucial for guiding the trainees to decide what went well and what needs to be improved. This will foster growth mindset that leads to the development of the professional identity [16].

### Post-simulation Activities

In this phase, the reflection journals and the feedback sheets serve to document the post-simulation experiences and performance insights. Such tools would aid the trainees to consolidate their learning experience and prepare for subsequent training sessions, i.e., the experiential learning [8, 19].

## Phase 4: Implementation

This phase reflects the executive recipe of the SBL training program for radiation oncology trainees. The current phase ensures the engagement and the effective learning as per the instructional strategies, aligned with the design and development phases.

### Logistics Management

The training is structured in such a manner that the pre-simulation briefings and debriefing periods are all done within the time widow of the training schedule. Each session is thoughtfully crafted to take care of space as well as technology and takes ownership of each participant’s availability. So while making use of things like treatment planning software and simulation labs, one can feel good about making the optimum use of these factors. Moreover, most importantly, time for the SBL should be ensured by the scheduling of the training time not to coincide with daily practice [20].

### Briefing (Pre-simulation)

This establishes a foundation for application. It constitutes an introduction of the objectives to be achieved during the session while emphasizing much of its critical safety policies that concerns the cancer and OAR definitions. This time is also for the case scenarios familiarization of the learners on cases like laryngeal cancer (Table 1). The remaining portion of this briefing prepares the learner for the expected tools and software associated with activities to come [21]. The instructor can effectively use the GAS model in this phase [15].

### Hands-On Activity

In this crucial phase of SBL, the learners should practice and develop the delineation skills of tumors and OARs, supervised by the facilitator(s) (Table 2). George and Doto’s model [22], combined with the SNAPPS framework [23], would embrace the critical thinking and problem-solving skills. The facilitator should assure the concepts of collaborative learning and PAL, and the phase development of practical experience. In this phase, the instructor/facilitator should recognize and apply the experiential learning cycle, as described by Kolb [19].

**Table 2:** Scoring rubric of OSCE

Assessment item	Performance indicator	Score 1 (needs improvement)	Score 2 (meets basic competency)	Score 3 (proficient)
Tumor delineation	Accuracy of delineation of the primary tumor (left vocal cord) and surrounding margins	Inaccurate delineation: poor identification of tumor margins, significant overlap with normal tissues	Partially accurate delineation: correct tumor location identified, but minor errors in margins or some overlap with healthy tissue	Accurate delineation: correctly identifies tumor and margins with no overlap on healthy tissues
Cervical lymph node delineation	Identification of involved cervical lymph nodes (left-sided)	Missed identification of cervical lymph nodes, or incorrect nodes delineated	Correct lymph nodes identified but minor inaccuracies in contouring margins	Precise delineation of involved cervical lymph nodes with accurate margins
OAR delineation (spinal cord, carotid arteries, parotid glands)	Accurate sparing of OARs during tumor delineation	Poor sparing: Significant overlap with critical structures	Adequate sparing but slight proximity to critical structures	Clear sparing: no overlap with OARs, appropriate distance maintained
Use of treatment planning software	Proficiency in utilizing software tools for contouring	Difficulties in navigating the software, incomplete contours	Basic navigation of software tools, minor issues with contouring	Fluent use of software with accurate contours and adjustments
Adherence to contouring protocols	Following the standardized contouring guidelines and safety protocols	Failed to follow protocols, missed critical steps in contouring	Followed most protocols with few deviations	Strict adherence to protocols, applied all steps correctly

### Debriefing

Following the simulation, a debriefing session using the PEARLS model [16] engages learners in reflective practice, which is critical for consolidating learning and planning future application. Such debriefing process targets the technical skills and the decision-making processes performance during the simulation. The facilitator role in this phase aim to guide such discussion and to ensure its alignment with the decided ILOs. Consequently, highlights the areas of improvement, along with the observed strengths [24].

### Monitoring and Adjustments

The effectiveness of the training is continuously monitored, throughout this phase. Adjustments are continuously effectuated based on learner feedback and facilitator observations, in order to enhance the training’s effectiveness [25].

### Facilitator Roles

The trained facilitators apply the SNAPPS and PEARLS models effectively. Thus, ensures the management of the dynamics of the SL environment, which will in return, fosters an atmosphere of learning transfer [26].

### Integration with Existing Processes

The training aligns with the existing educational frameworks and the day-to-day practice of the institution. This integration confirms that the SBL training is not an isolated event but a cohesive part of the broader educational goals. This will enhance its authenticity and applicability to clinical practice [27].

## Phase 5: Evaluation: The Kirkpatrick’s Model

Generally, the Kirkpatrick’s model is a standard for evaluation of the outcome of training programs. Consequently, we used the Kirkpatrick model to evaluate the radiation oncology delineation skills simulation, across its four levels—reaction, learning, behavior, and results. Such approach ensures a thorough understanding of the educational impact and clinical relevance of the SBL training program (Table 3) [28–30].

**Table 3:** Assessment items and tools in Kirkpatrick’s model framework

Level	Evaluation focus	Assessment method	Metrics	Who completes it
Level 1: Reaction	Learners’ satisfaction and engagement	Post-simulation survey (Likert scale, open-ended questions)	Satisfaction score (1–5), qualitative feedback	Simulation participants
Level 2: Learning	Knowledge and skills acquisition	OSCE with a scoring rubric (tumor, lymph node, OAR delineation)	1–3 rating on each task (needs improvement, meets expectations, proficient)	Two independent raters (faculty)
Level 3: Behavior	Application of skills in practice	Formative assessments, longitudinal follow-up in clinical settings	Yes/no scale for protocol adherence, clinical observations	Clinical supervisors and facilitators
Level 4: Results	Impact on clinical outcomes	Post-course audits, patient outcome tracking	Error rates in treatment planning, patient complication rates	Quality improvement teams, faculty

### Validity and Reliability

We assured the validation and reliability of assessments methods, by aligning the OSPE items with our decided learning objectives and the instructional materials and methods (e.g., triangulation [31].

## Conclusion

Employing the ADDIE model within simulation-based learning (SBL) frameworks effectively meets the intricate educational needs in radiation oncology training. By systematically applying each phase—analysis, design, development, implementation, and evaluation—this model ensures that training programs are meticulously tailored to enhance clinical performance.
